# A nullcline-guided discrete-time map for neurons with subcritical Hopf bifurcation dynamics

**DOI:** 10.3389/fncom.2026.1857116

**Published:** 2026-07-23

**Authors:** Mustafa Zeki, Sinan Kapçak, Hunseok Kang

**Affiliations:** 1 College of Engineering and Technology, American University of the Middle East, Egaila, Kuwait; 2 Department of Mathematics and Computer Science, Eindhoven University of Technology, Eindhoven, Netherlands

**Keywords:** bistability, discrete-time dynamical system, resonance, single-neuron modeling, subcritical Hopf bifurcation

## Abstract

Neurons near a subcritical Hopf bifurcation are distinguished by their ability to produce subthreshold oscillations, maintain bistability between rest and repetitive spiking, and fire preferentially in response to inputs near their intrinsic resonant frequency. Simulating these properties in large neural networks using continuous-time models such as the Hodgkin-Huxley formalism is computationally prohibitive, motivating the search for reduced representations that retain the essential dynamics. Here we derive a two-dimensional discrete-time map from the continuous *I*_*Na, p*_+*I*_*K*_ model by exploiting the time-scale separation between the membrane potential and the potassium gating variable, and using nullcline geometry to approximate the slow drift along each branch of the cubic *v*-nullcline. The resulting map consists of an outer piecewise-linear loop that produces realistic action potential waveforms and an inner switching region that reproduces focus-like subthreshold oscillations, with a slow recovery variable governing the transition between the two. The injected current modulates the size of the inner region, naturally encoding the bifurcation structure of the original system. Numerical simulations confirm that the discrete model reproduces the main dynamical signatures of bistability, hysteresis under a slowly varying current ramp, and frequency-selective firing in response to periodic burst stimulation—all without solving a single differential equation. Crucially, the map retains selected physically interpretable parameters of the continuous model while replacing continuous-time integration with a lightweight algebraic update. Benchmarking against explicit Euler and fourth-order Runge–Kutta integration shows that the optimized map has a per-update cost comparable to Euler and substantially lower than Runge–Kutta, supporting its use as a compact, interpretable discrete representation of subcritical-Hopf dynamics rather than as a direct numerical integrator of the continuous model.

## 1 Introduction

The dynamics of individual neurons are most faithfully described by conductance-based models in the Hodgkin-Huxley tradition (Ermentrout and Terman, 2010b; Izhikevich, 2007; Nelson and Rinzel, 1998), in which the membrane potential and one or more gating variables evolve according to coupled nonlinear ordinary differential equations. Although these models capture a broad range of electrophysiological phenomena, their computational cost is substantial: even a single neuron typically requires four state variables, and networks of biologically realistic size involve tens of thousands of coupled equations that must be integrated repeatedly over many parameter settings (Ermentrout and Terman, 2010a; Gerstner and Kistler, 2002). This overhead makes large-scale parameter exploration impractical with full conductance-based frameworks.

Several strategies have been developed to address this burden. Integrate-and-fire models replace the continuous spike-generation mechanism with a threshold-and-reset rule, achieving dramatic speed gains at the cost of detailed spike shape and subthreshold dynamics (Burkitt, 2006; Zeki and Balcı, 2019). A second class retains differential-equation structure while reducing the number of variables, exploiting either time-scale separation, nullcline analysis, and geometric singular perturbation ideas (Fenichel, 1979; Rubin and Terman, 2002), or algebraic simplification of channel kinetics (FitzHugh, 1955; Morris and Lecar, 1981; Kepler et al., 1992; Krinskii and Kokoz, 1973). Representative examples include the FitzHugh–Nagumo and Morris–Lecar models. The simple two-variable model of Izhikevich (2003) pushes this reduction further, reproducing a wide catalog of firing patterns through two coupled ordinary differential equations and an after-spike reset rule. Its parameters are effective reduced-model quantities rather than direct representations of specific ionic conductances.

A third approach replaces continuous time with discrete iteration, constructing a map whose iterates approximate the trajectory of the original system. Early work in this direction used Poincaré section techniques, sampling continuous oscillations at a fixed phase to obtain a one-dimensional return map (Channell et al., 2007). Terman et al. (2008) extended this to study synchronization in networks of bursting neurons. Although efficient, Poincaré-based maps are intrinsically tied to periodic solutions and cannot represent transient dynamics, threshold crossings from rest, or subthreshold behavior. Phenomenological two-dimensional maps offer a complementary approach: by choosing nonlinear functions with suitable bifurcation properties, one can reproduce many firing patterns without reference to ion channels. Influential examples include the models of Chialvo (1995) and Rulkov (2002), and comprehensive surveys of this map-based literature are provided by Ibarz et al. (2011) and Girardi-Schappo et al. (2013). The strength of these models is simplicity; the limitation is that their parameters are not directly tied to measurable biophysical quantities such as maximal conductances or reversal potentials.

In our previous work (Zeki and Kapçak, 2023), we developed a discrete-time map for neurons exhibiting a saddle-node on invariant circle (SNIC) bifurcation. The central idea was to embed the nullcline geometry of the continuous phase plane directly into the map rule: the slow drift along each branch of the cubic *v*-nullcline is approximated by a nullcline-guided increment, so that the maximal conductances, reversal potentials, and membrane capacitance of the full model all appear explicitly in the discrete update. The resulting 1-D map reproduces threshold dynamics and the zero-onset current-frequency (*I*-*F*) relationship of type-I neurons, while a 2-D extension captures spike-frequency adaptation. Unlike Poincaré map approaches, the map operates throughout the cycle and describes both subthreshold transients and sustained oscillations within a single framework.

The present paper extends this programme to a qualitatively different class of neurons: those whose transition from rest to repetitive firing occurs through a subcritical Hopf bifurcation. Such neurons, often called *resonators* (Izhikevich, 2000; Hutcheon and Yarom, 2000), differ fundamentally from SNIC-type *integrators* in their subthreshold behavior. Near the resting state, small perturbations do not decay monotonically; instead, the equilibrium is a stable focus enclosed by an unstable limit cycle, so perturbations return to rest through damped oscillations at a characteristic frequency (Izhikevich, 2007; Rinzel and Ermentrout, 1989). This confers a preferred input frequency: stimuli delivered in phase with the intrinsic subthreshold oscillation accumulate across cycles and can drive the trajectory across the unstable limit cycle into a large-amplitude spiking state, whereas off-frequency inputs do not (Hutcheon and Yarom, 2000; Izhikevich, 2001). The same bistable structure produces hysteresis: once spiking is initiated, reducing the injected current does not necessarily return the neuron to rest, because the spiking orbit and the resting state coexist over a range of currents. These properties—resonance and hysteresis—have no analog in integrator-type neurons and confer distinct computational advantages for frequency-coded information processing (Izhikevich, 2007). Experimentally, resonator-type excitability is observed in neurons of the entorhinal cortex, thalamus, inferior olive, and auditory brainstem, among others (Hutcheon and Yarom, 2000).

### 1.1 Positioning relative to existing reduced and discrete neuron models

The present construction differs from existing reduced and discrete approaches primarily in the relationship between the map structure and the underlying conductance-based model. Unlike integrate-and-fire models and the two-variable threshold–reset model of Izhikevich (2003), the proposed map is designed to represent an explicit full-cycle voltage loop, including the subthreshold return toward rest and the large-amplitude spiking trajectory. Unlike Poincar'e-section maps (Channell et al., 2007; Terman et al., 2008), it is defined throughout the voltage range and is not restricted to sampling an already established periodic orbit. Unlike phenomenological maps such as those of Chialvo (1995) and Rulkov (2002), whose nonlinearities are selected to reproduce a broad repertoire of excitable, spiking, and bursting dynamics, the slow branches and outer-loop bounds of the present map are guided by the phase-plane geometry of the continuous *I*_*Na, p*_+*I*_*K*_ model. More specifically, the *v*-nullcline and the limiting approximations to *n*_∞_(*V*) on the slow branches motivate the slow-drift terms of the map. Consequently, changes in the maximal conductances and reversal potentials modify selected map components in a physiologically interpretable manner.

The closest existing reduced model in spirit is the resonate-and-fire neuron (Izhikevich, 2001), which is likewise organized around subthreshold focus dynamics and captures frequency preference efficiently. It differs from the present construction in being linear below threshold, representing spike generation through a threshold-and-reset rule, and using effective linear coefficients rather than ionic conductances and reversal potentials. At the same time, the proposed map should not be interpreted as a fully parameter-free reduction: the inner switching region and selected waveform-shaping coefficients remain phenomenological, as discussed in Section 2.4. Its distinctive contribution is therefore a nullcline-guided discrete framework that combines selected conductance-linked structure with a representation of the focus-like subthreshold oscillations, bistability, hysteresis, and resonance associated with subcritical Hopf excitability.

Other reduced families share individual features of the present construction but differ in scope. Theta-neuron and quadratic integrate-and-fire reductions (Ermentrout, 1996; Latham et al., 2000) are canonical normal forms for type-I (integrator) excitability arising through a saddle-node-on-invariant-circle bifurcation; in their standard one-dimensional form they do not represent the type-II resonant subthreshold dynamics that characterize the Hopf regime treated here, unless extended with an additional recovery variable. Two-dimensional subthreshold-resonance models (Rotstein and Nadim, 2014) analyse frequency preference directly in terms of interacting resonant and amplifying currents, but remain continuous-time differential equations. Piecewise-linear caricatures of excitable systems, such as the McKean (1970) reduction of the FitzHugh–Nagumo model, likewise simplify the vector field while preserving phase-plane interpretability, yet they too are integrated in continuous time. The present construction differs from all of these in formulating the reduced dynamics directly as an algebraic discrete-time iteration: it retains nullcline-guided geometric interpretability while replacing continuous-time integration with a low-cost update that reproduces the coexistence of rest, subthreshold oscillation, large-amplitude spiking, bistability, and hysteresis specific to the post-Hopf bistable regime.

To our knowledge, no discrete-time map has been proposed that preserves the nullcline-based parameters of a subcritical Hopf neuron and simultaneously represents subthreshold oscillations, bistability, hysteresis, and resonance. Here we fill that gap. Building on the framework of Zeki and Kapçak (2023), we construct a two-dimensional piecewise map from the *I*_*Na, p*_+*I*_*K*_ model (Izhikevich, 2007) in which an outer spiking loop and an inner focus region are separated by a current-dependent switching boundary. We demonstrate that the resulting map reproduces all three hallmark properties of subcritical Hopf excitability without solving any differential equation.

## 2 Materials and methods

### 2.1 Mathematical model for subcritical Hopf bifurcation dynamics

#### 2.1.1 Model

To simulate the dynamics of subcritical Hopf bifurcation in an individual neuron with respect to the injected current parameter, we employ the *I*_*Na, p*_+*I*_*K*_ model as described by Izhikevich (2007). This model incorporates three intrinsic currents: the leak current, the persistent sodium current, and the potassium current. The activation of the sodium gate is assumed to occur instantaneously. The model consists of two state variables: the membrane potential (*V*) and the potassium gating variable (*n*). The governing differential equations (Equations 1, 2) are:


$$\begin{array}{l} C\frac{dV}{dt}=I-g_{L}\left(V-E_{L}\right)-g_{Na}m_{\infty }\left(V\right)\left(V-E_{Na}\right)\\ \;-g_{K}n\left(V-E_{K}\right), \end{array}$$ (1)

$$\begin{array}{l} \frac{dn}{dt}=\varepsilon \frac{n_{\infty }\left(V\right)-n}{\tau \left(V\right)}. \end{array}$$ (2)

The maximal conductances are $g_{L}=1{\text{mS/cm}}^{2}$, $g_{Na}=4{\text{mS/cm}}^{2}$, and $g_{K}=4{\text{mS/cm}}^{2}$. The membrane capacitance is *C* = 1μF/cm^2^. The reversal potentials are *E*_*L*_ = −78mV, *E*_*Na*_ = 60mV, and *E*_*K*_ = −90mV.

The steady-state activation functions *m*_∞_(*V*) and *n*_∞_(*V*) are Boltzmann functions of the form (Equation 3):


$$\begin{array}{l} B\left(V\right)=\frac{1}{1+\exp\left(\frac{V_{1/2}-V}{k}\right)}. \end{array}$$ (3)

For *m*_∞_(*V*), the midpoint and slope are *V*_1/2_ = −30mV and *k* = 7; for *n*_∞_(*V*), they are *V*_1/2_ = −45mV and *k* = 5. Following the *I*_Na, p_+*I*_*K*_ parameterisation of Izhikevich (2007), the potassium time constant is taken to be constant, τ(*V*) = 1. In this minimal form, the recovery time scale is carried explicitly by ε rather than by a voltage-dependent τ_*n*_(*V*). Because τ enters the rate of approach to the *n*-nullcline but not the nullcline equations themselves, the constant choice τ(*V*) = 1 does not change the locations of the nullcline intersections. However, voltage-dependent recovery kinetics can affect quantitative stability properties, bifurcation thresholds, oscillation periods, and the precise width of the bistable regime. The bifurcation values and dynamical comparisons reported below should therefore be interpreted for the constant-τ parameterisation used in this study. The complete parameter set used throughout the study is summarized in Table 1.

**Table 1:** Complete parameter set used to reproduce all figures.

Symbol	Value	Description
*C*	1μF/cm^2^	Membrane capacitance
*g*_*L*_	1mS/cm^2^	Leak conductance
*g*_*Na*_	4mS/cm^2^	Sodium conductance
*g*_*K*_	4mS/cm^2^	Potassium conductance
*E*_*L*_	−78mV	Leak reversal potential
*E*_*Na*_	60mV	Sodium reversal potential
*E*_*K*_	−90mV	Potassium reversal potential
*V*_1/2, *m*_, *k*_*m*_	−30mV, 7	*m*_∞_ midpoint, slope
*V*_1/2, *n*_, *k*_*n*_	−45mV, 5	*n*_∞_ midpoint, slope
τ	1 (constant)	Potassium time constant
ε	0.5	Time-scale separation
Discrete-map geometry (at applied current *I*)		
*I*_low_, *I*_high_	32, 36μA	Bistable-window bounds
*w*_0_	8	Inner-region width scale
δ_*v*_	*w*_0_(*I*_high_−*I*)/(*I*_high_−*I*_low_)	Current-dependent half-width
*c*_1_	3	Inner-region extent multiple
(*v*_0_, *n*_0_)	nullcline intersection	Resting equilibrium at *I*
(*k*_*r*0_, *k*_*r*1_)	*v*-nullcline knees	Outer switching thresholds

Continuous-model parameters define the *I*_Na, p_+*I*_*K*_ system; the discrete-map geometry parameters define the inner region and switching bounds at a given applied current *I*. The applied current *I* is the bifurcation parameter and is varied across the bistable window (figure-specific values given in the captions).

### 2.2 Subcritical Hopf bifurcation in the continuous model

Bifurcations provide the mathematical framework for understanding how neurons transition from rest to repetitive spiking (Rinzel and Ermentrout, 1989; Izhikevich, 2007). In this work we examine the subcritical Hopf bifurcation using the *I*_*Na, p*_+*I*_*K*_ model. The phase-plane interpretation, including the use of voltage and recovery nullclines to classify equilibria, limit cycles, and bifurcation structure, follows the standard geometric framework for conductance-based neuron models (Rinzel and Ermentrout, 1989; Ermentrout and Terman, 2010a; Izhikevich, 2007).

**Figure 1:**
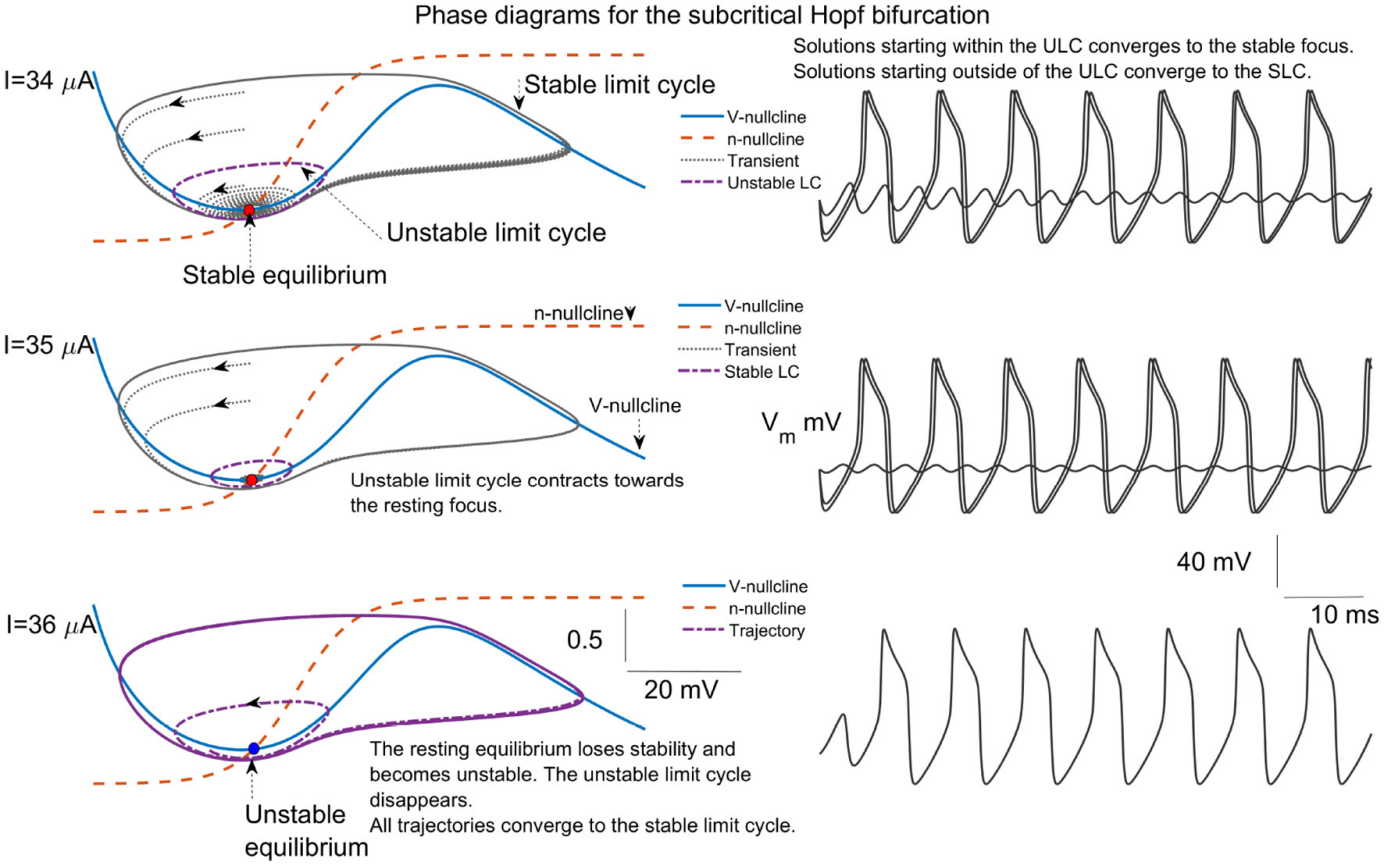
Phase-space evolution of the continuous *I*_*Na, p*_+*I*_*K*_ model for increasing applied current. All panels use *C* = 1μF/cm^2^, $g_{L}=1\text{mS}/\text{c}{\text{m}}^{2}$, $g_{Na}=4\text{mS}/\text{c}{\text{m}}^{2}$, $g_{K}=4\text{mS}/\text{c}{\text{m}}^{2}$, *E*_*L*_ = −78mV, *E*_*Na*_ = 60mV, *E*_*K*_ = −90mV, *m*_∞_ midpoint −30mV with slope 7, and *n*_∞_ midpoint −45mV with slope 5, and recovery rate ε = 0.5. The **(top)** and **(middle)** panels show the bistable regime at representative currents *I* = 34μA and *I* = 35μA, where a stable equilibrium and a stable limit cycle (SLC) are separated by an unstable limit cycle (ULC). The **(bottom)** panel shows the post-transition regime at *I* = 36μA, where the resting equilibrium has lost stability.

**Figure 2:**
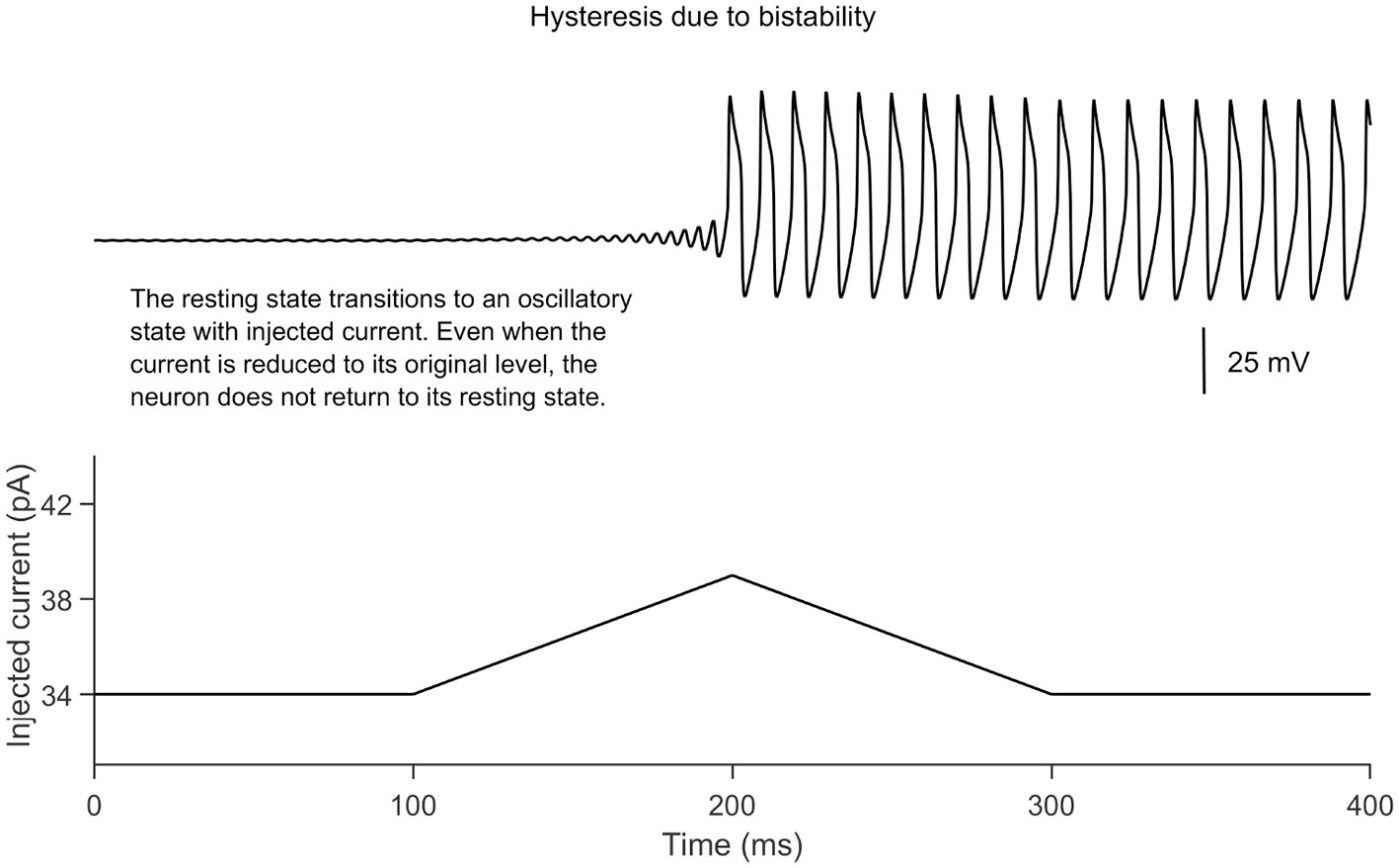
Hysteresis in the continuous *I*_*Na, p*_+*I*_*K*_ model using the same conductance and activation parameters as in Figure 1. The injected current is slowly increased and then decreased back toward its baseline value. Because the stable resting state and the stable spiking limit cycle coexist over part of the current range, the trajectory remains in the oscillatory state even after the current is reduced.

**Figure 3:**
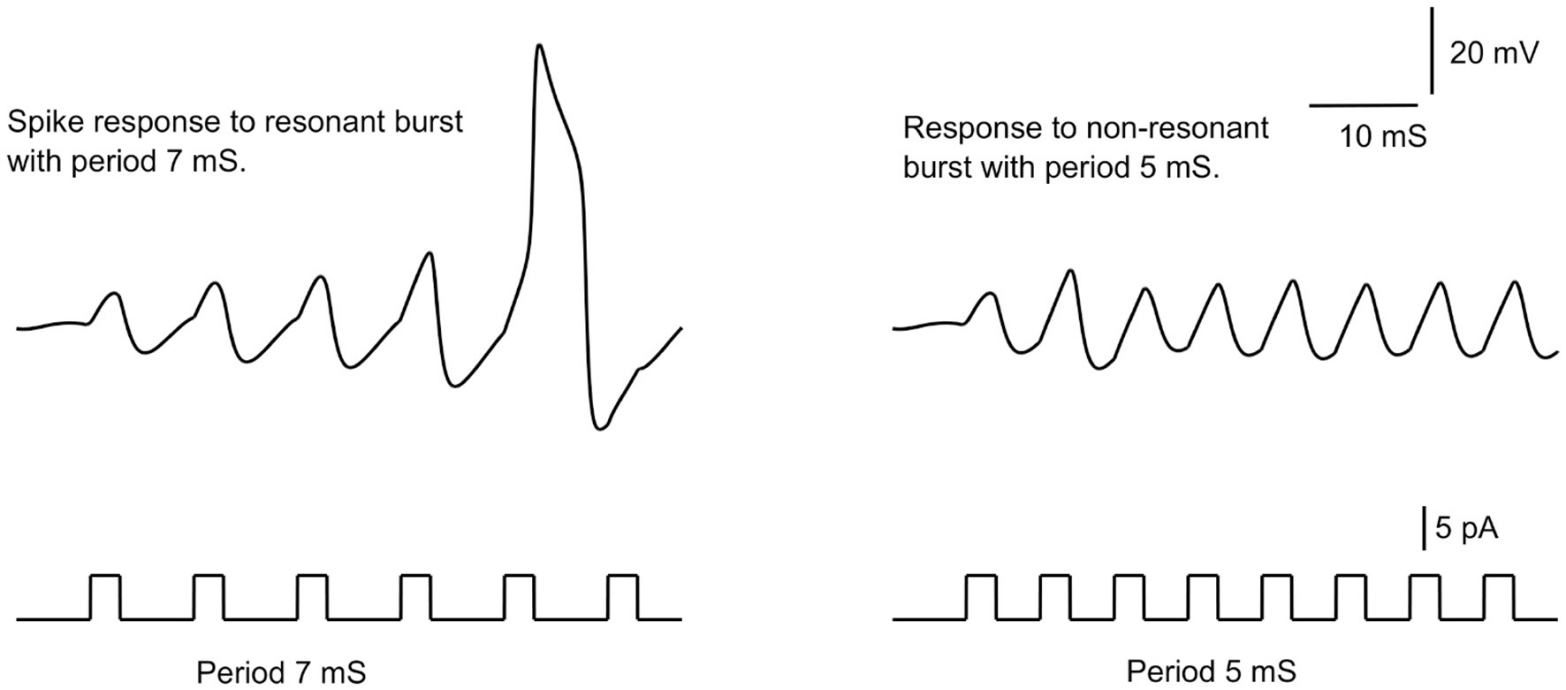
Frequency-selective response of the continuous *I*_*Na, p*_+*I*_*K*_ model using the same conductance and activation parameters as in Figure 1. A burst input with period 7ms **(left)** drives the resting focus into the spiking regime, whereas a burst with period 5ms **(right)** does not, illustrating the frequency selectivity of the bistable regime.

Figures 1–3 reproduce the established behavior of the continuous *I*_*Na, p*_+*I*_*K*_ model and are not presented as new results. They provide the reference dynamics against which the discrete map is compared. The trajectories were generated in MATLAB using the ode45 solver, an adaptive explicit Runge–Kutta integrator based on the Dormand–Prince (4, 5) pair, with the default error tolerances (relative 10^−3^, absolute 10^−6^), from representative initial conditions appropriate to the illustrated dynamical regimes. The *v*- and *n*-nullclines in Figure 1 were plotted directly from their defining equations. The parameter values used in the simulations are those stated above.

The defining characteristic of this bifurcation is the presence of a bistable regime (Figure 1). At *I* = 34μA, the system has a stable equilibrium enclosed by an unstable limit cycle (ULC), which is itself enclosed by a larger stable limit cycle (SLC). The ULC acts as a *separatrix*: trajectories inside its boundary converge to the resting focus, while those outside are attracted to the SLC and produce periodic spiking. As the current increases to 35μA, the ULC contracts toward the resting focus. At *I* = 36μA the ULC collapses into the resting equilibrium, which then loses stability, and all trajectories converge to the SLC.

#### 2.2.1 Local stability of the resting equilibrium

The Hopf transition can be characterized analytically by linearising the continuous model about its equilibrium. Let


$$P\left(V,n\right)=I-g_{L}\left(V-E_{L}\right)-g_{Na}m_{\infty }\left(V\right)\left(V-E_{Na}\right)-g_{K}n\left(V-E_{K}\right)$$

and


$$Q\left(V,n\right)=\varepsilon \frac{n_{\infty }\left(V\right)-n}{\tau \left(V\right)}.$$

Thus the continuous vector field is


$$\frac{dV}{dt}=\frac{1}{C}P\left(V,n\right),\;\frac{dn}{dt}=Q\left(V,n\right).$$

An equilibrium (V_*_, n_*_) satisfies *P*(V_*_, n_*_) = 0 and *Q*(V_*_, n_*_) = 0, equivalently n_*_ = *n*_∞_(V_*_). Its Jacobian is


$$\begin{array}{l} J=\left(\begin{array}{cc} \frac{1}{C}\left[-g_{L}-g_{Na}(m_{\infty }^{\prime }\left(V_{*}\right)\left(V_{*}-E_{Na}\right)+m_{\infty }\left(V_{*}\right))-g_{K}n_{*}\right] & -\frac{g_{K}}{C}\left(V_{*}-E_{K}\right)\\ \frac{\varepsilon }{\tau }n_{\infty }^{\prime }\left(V_{*}\right) & -\frac{\varepsilon }{\tau } \end{array}\right), \end{array}$$ (4)

where $m_{\infty }^{\prime }\left(V\right)=m_{\infty }\left(V\right)\left(1-m_{\infty }\left(V\right)\right)/k_{m}$, $n_{\infty }^{\prime }\left(V\right)=n_{\infty }\left(V\right)\left(1-n_{\infty }\left(V\right)\right)/k_{n}$, and, in the present simulations, *C* = 1, τ = 1, and ε = 0.5.

The equilibrium is a stable focus when tr*J* < 0 and (tr*J*)^2^ < 4det*J*, and it loses stability through a Hopf transition when tr*J* = 0 with det*J*>0. Evaluating Equation 4 along the resting branch (V_*_ ≈ −53 mV) gives a stable focus with eigenvalues λ_1, 2_ = −0.027 ± 1.37*i* at *I* = 34μA and λ_1, 2_ = −0.008 ± 1.40*i* at *I* = 35μA. The real part vanishes at approximately *I*_Hopf_ ≈ 35.4μA, where λ_1, 2_ ≈ ±1.41*i*. Beyond this point the equilibrium is an unstable focus; for example, λ_1, 2_ = 0.012 ± 1.42*i* at *I* = 36μA. This local eigenvalue behavior supports the Hopf stability change shown in Figure 1. Together with the phase-plane coexistence of a stable resting focus, an unstable separating limit cycle, and a stable spiking limit cycle, it provides quantitative evidence for the subcritical Hopf structure used as the reference dynamics for the discrete map.

This bistable structure leads to hysteresis (Figure 2): because the SLC and the resting state coexist over a range of currents, the neuron's state depends on its history. Ramping the current up drives the neuron into spiking; reducing the current back to its original level does not return the neuron to rest, because the trajectory is trapped within the basin of the SLC.

Beyond thresholding, the stable focus near the resting state produces damped oscillations when perturbed. As shown in Figure 3, this gives the neuron resonator properties (Hutcheon and Yarom, 2000; Izhikevich, 2001): a burst input with a 7 ms period matches the intrinsic subthreshold rhythm and accumulates across cycles until the ULC is crossed and a spike fires, whereas a 5 ms burst fails to produce sufficient build-up and prevents spiking. This frequency selectivity fundamentally distinguishes resonator neurons from integrator-type neurons, which respond preferentially to stronger rather than better-timed inputs (Izhikevich, 2000).

### 2.3 A conceptual prototype for subcritical-Hopf-like bistability

Before constructing the full nullcline-guided neuronal map, we introduce a dimensionless prototype that serves a purely pedagogical purpose: to illustrate, in the simplest possible discrete-time setting, the qualitative attractor geometry required to represent subcritical-Hopf-like bistability. This prototype is not intended as a general neuron model, is not derived directly from the continuous *I*_*Na, p*_+*I*_*K*_ system, and is not used as the update rule of the final map. Its purpose is narrower: to show how a stable fixed point, an unstable separating period-2 orbit, and a larger stable period-2 orbit can coexist within a simple discrete framework. The variable *x* is dimensionless and has no direct biophysical interpretation.

#### 2.3.1 Map construction and period-2 solutions

The purpose of the prototype is to reproduce, in the simplest possible discrete setting, the qualitative attractor structure associated with the bistable regime of a subcritical Hopf neuron. In a one-dimensional map *x*_*n*+1_ = *f*(*x*_*n*_), a fixed point x_*_ satisfies *f*(x_*_) = x_*_. A period-2 orbit is a pair of distinct points {x_*_, *f*(x_*_)} that return to their initial values after two iterations; equivalently, each point of a period-2 orbit satisfies Equation 5:


$$\begin{array}{l} f^{2}\left(x_{*}\right)=f\left(f\left(x_{*}\right)\right)=x_{*},\;f\left(x_{*}\right)\neq x_{*}. \end{array}$$ (5)

These are standard definitions for discrete dynamical systems (Elaydi, 2005).

For an odd function, *f*(−*x*) = −*f*(*x*), a symmetric period-2 orbit {x_*_, −x_*_} can be identified from intersections of the map with *y* = −*x*: if *f*(x_*_) = −x_*_, then $f^{2}\left(x_{*}\right)=f\left(-x_{*}\right)=-f\left(x_{*}\right)=x_{*}$. In the present conceptual prototype, the stable fixed point at the origin represents the resting state, the intermediate unstable period-2 orbit represents a separating boundary between the resting and active regimes, and the larger stable period-2 orbit provides a minimal recurrent analog of the spiking state. This interpretation is qualitative: the variable *x* is dimensionless and is not identified directly with membrane voltage or any other biophysical variable.

#### 2.3.2 Local stability of the prototype period-2 orbits

The local stability of a period-2 orbit is determined by the multiplier of the second iterate. For a period-2 point x_*_,


$${\left(f^{2}\right)}^{\prime }\left(x_{*}\right)=f^{\prime }\left(f\left(x_{*}\right)\right)f^{\prime }\left(x_{*}\right).$$

The orbit is locally stable if $|{\left(f^{2}\right)}^{\prime }\left(x_{*}\right)|<1$ and unstable if $|{\left(f^{2}\right)}^{\prime }\left(x_{*}\right)|>1$ (Elaydi, 2005). For the symmetric period-2 orbit considered here, *f*(x_*_) = −x_*_; since the derivative of an odd function is even, $f^{\prime }\left(-x_{*}\right)=f^{\prime }\left(x_{*}\right)$, and the condition reduces to


$$|{\left[f^{\prime }\left(x_{*}\right)\right]}^{2}|<1.$$

This calculation is used only to classify the fixed point and periodic orbits of the dimensionless prototype; it is not a stability analysis of the full nullcline-guided neuronal map of Section 2.4. Applied here, it confirms that the resting fixed point and the spiking period-2 orbit are simultaneously stable while the intermediate period-2 orbit is unstable, reproducing in discrete time the bistable organization of the continuous *I*_*Na, p*_+*I*_*K*_ model (Figure 1).

#### 2.3.3 Parameter control and bifurcation behavior

**Figure 4:**
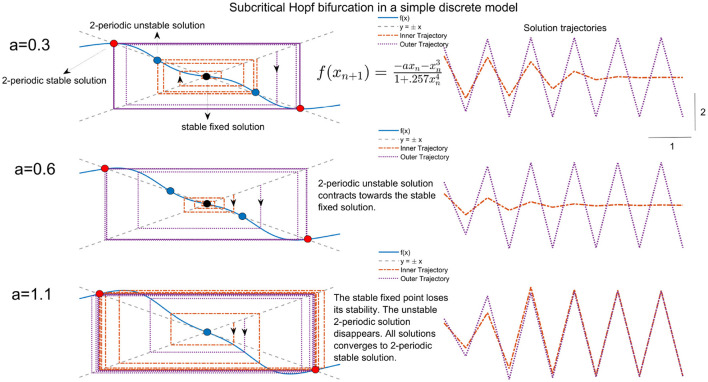
Phase-plane analysis and solution trajectories for the dimensionless conceptual prototype $x_{n+1}=\left(-ax_{n}-x_{n}^{3}\right)/\left(1+0.257x_{n}^{4}\right)$. **(Top)**, *a* = 0.3, showing bistability between the stable origin and a large-amplitude stable period-2 orbit. **(Middle)**, *a* = 0.6, showing contraction of the unstable period-2 boundary. **(Bottom)**, *a* = 1.1, showing loss of stability of the origin and convergence to the large stable period-2 orbit. The variable *x* is dimensionless and is not identified with membrane voltage.

**Figure 5:**
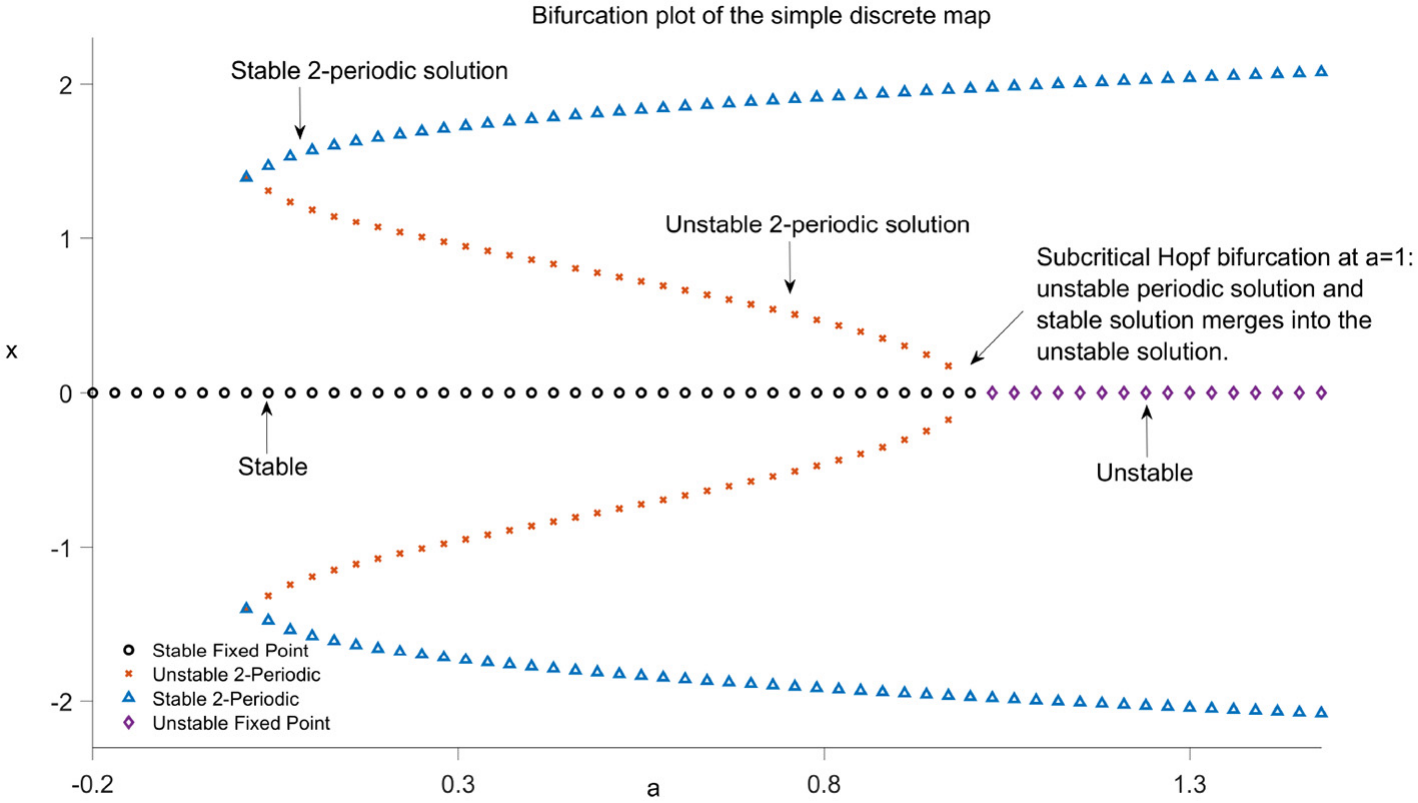
Global bifurcation diagram of the dimensionless conceptual prototype $x_{n+1}=\left(-ax_{n}-x_{n}^{3}\right)/\left(1+0.257x_{n}^{4}\right)$. The horizontal axis is the prototype control parameter *a*. The merging of the stable origin and the unstable period-2 branch near *a* ≈ 1 characterizes the subcritical-Hopf-like transition in the prototype.

**Figure 6:**
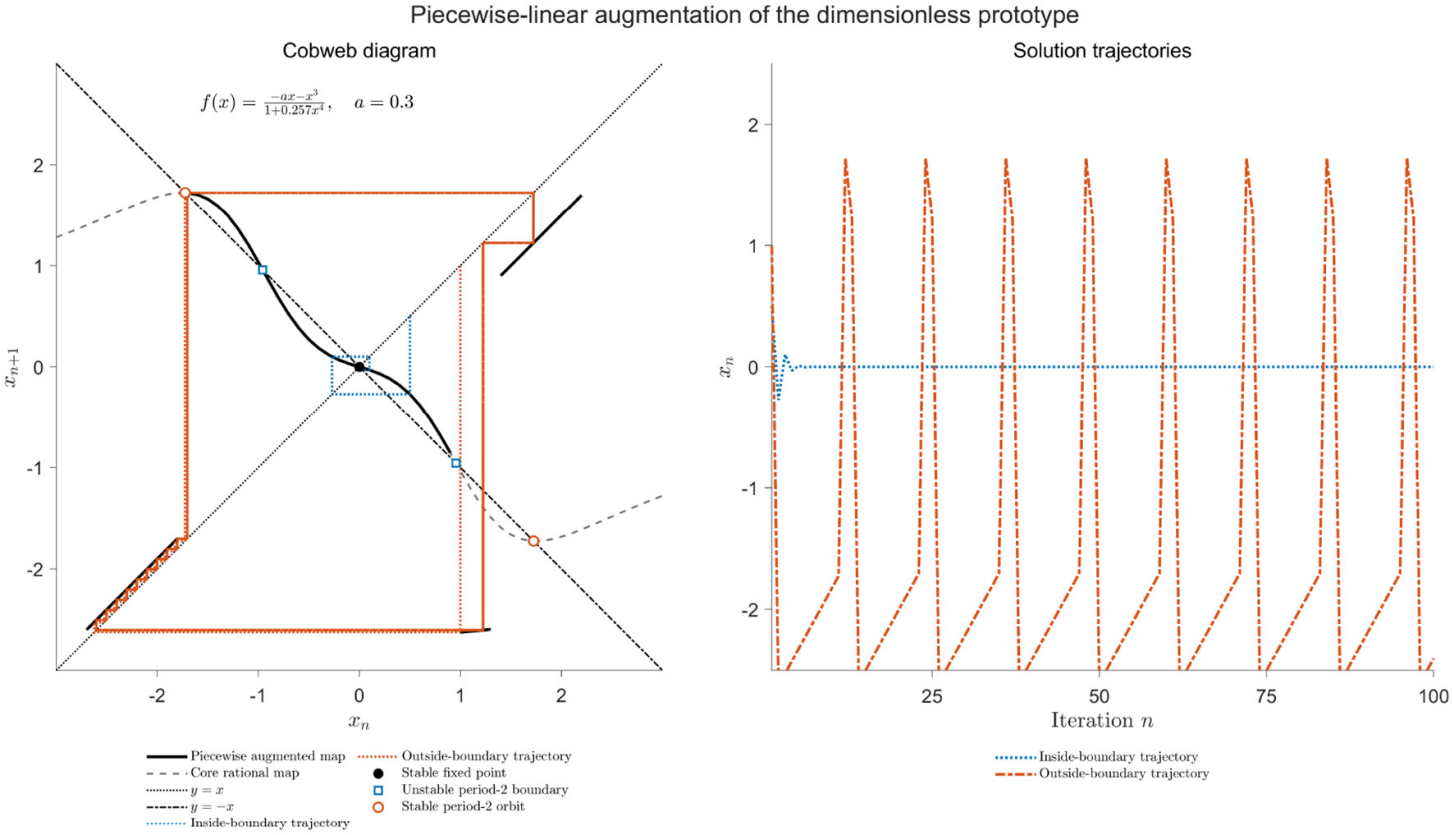
Piecewise-linear augmentation of the dimensionless conceptual prototype. The cobweb diagram **(left)** shows how the core rational map with *a* = 0.3 is augmented by piecewise-linear segments to shape the large-amplitude stable orbit. The trajectories **(right)** compare damped subthreshold-like oscillations inside the unstable period-2 boundary with the relaxation-style large-amplitude orbit. The variables in this prototype are dimensionless and serve only as a conceptual bridge to the full neuronal map.

Figures 4–6 were generated directly from the dimensionless prototype of Equation 6 and its piecewise-linear augmentation, not from the continuous *I*_*Na, p*_+*I*_*K*_ model. Figure 4 was obtained by direct iteration of the prototype at the representative control values *a* = 0.3, *a* = 0.6, and *a* = 1.1, with initial conditions taken both inside and outside the unstable period-2 boundary; the fixed point and period-2 orbits were located from the intersections of *f*(*x*) with *x* and −*x*, and classified using the second-iterate multiplier introduced above. Figure 5 was obtained by sweeping the same control parameter *a* and plotting the resulting stable and unstable branches. Figure 6 was generated from the same core prototype after adding the piecewise-linear segments used only to shape the large-amplitude recurrent orbit. These figures are conceptual illustrations of the attractor geometry that motivates the full neuronal map; they are not simulations of the continuous model.

For this conceptual illustration, we consider the following odd rational map:


$$\begin{array}{l} x_{n+1}=f\left(x_{n}\right)=\frac{-ax_{n}-x_{n}^{3}}{1+0.257x_{n}^{4}}, \end{array}$$ (6)

where *a* controls the distance between the origin and the unstable 2-periodic solution. The coefficient 0.257 is a shaping parameter selected to produce a simple saturating prototype with the required qualitative geometry. Neither *a* nor the coefficient 0.257 is interpreted as a biophysical parameter of the continuous neuron model.

The qualitative behavior under varying *a* is illustrated in Figure 4. At *a* = 0.3 the system is bistable: the stable origin (black dot) is surrounded by an unstable period-2 orbit (blue) which is enclosed by a stable period-2 orbit (red), providing a direct discrete analog of the continuous bistable regime. As *a* increases, the unstable orbit contracts toward the origin.

The bifurcation diagram (Figure 5) shows that at the critical threshold (*a* = 1.1) the unstable orbit collides with the origin, which then becomes unstable, leaving the stable 2-periodic solution as the sole attractor. This sequence is the discrete analog of the subcritical Hopf bifurcation in continuous-time systems (Izhikevich, 2007; Rinzel and Ermentrout, 1989).

#### 2.3.4 Trajectory shaping via piecewise linear augmentation

The cubic rational map captures the essential bifurcation structure, but the resulting trajectories lack the characteristic profile of neuronal action potentials. We enrich the map by incorporating piecewise linear components (Figure 6).

The core dynamics near the origin are governed by $f\left(x\right)=\frac{-0.3x-x^{3}}{1+0.257x^{4}}$, preserving the stable fixed point. Initial conditions within the unstable period-2 boundary return to rest via damped focus-like oscillations (blue trajectory), mimicking subthreshold resonator behavior. Piecewise linear segments at larger amplitudes define the large-amplitude spiking orbit (red loop), producing relaxation-style action potentials with distinct active and silent phases.

The prototype in Equation 6 provides only a conceptual motivation for the architecture of the neuronal map constructed below. There is no one-to-one identification between the dimensionless variable *x* and the biophysical variables (*v, z*), and the rational function *f*(*x*) is not used directly as the inner update *F*_in_(*v, z*). In the full model, *v* represents membrane potential, *z* is an auxiliary recovery variable, the outer loop is defined by the nullcline-guided branches in Equation 16, and the inner switching region is represented separately by Equation 17.

### 2.4 Construction of the discrete map

The discrete model is constructed only for the post-crossing bistable regime, *v*_0_>*k*_*r*0_, where the continuous system has coexisting rest and spiking states. The same nullcline-guided approach used for SNIC neurons (Zeki and Kapçak, 2023) is applied here, extended with an inner switching region that captures the focus dynamics specific to subcritical Hopf excitability. The map is a switching system with an outer loop for spiking and an inner region for subthreshold oscillations, with a recovery variable *z*_*n*_ modulating the boundary between the two. The construction rests on the same time-scale-separation assumption as the continuous reduction: the membrane potential *v* evolves much faster than the recovery variable, quantified by a small parameter ε≪1, so that slow motion proceeds along the *v*-nullcline.

For clarity, the construction is presented in five steps. First, the *v*-nullcline of the continuous *I*_*Na, p*_+*I*_*K*_ model is used to define the slow-drift terms (Step 1). Second, the voltage bounds *v*_low_ and *v*_high_ of the outer spiking loop are estimated from limiting conductance balances (Step 2). Third, the current-dependent inner region Ω_in_(*I*) is defined to represent the basin of the resting focus in the bistable regime (Step 3). Fourth, the full update is assembled as a switching system and the outer update *F*_out_ is specified for trajectories outside the inner region (Step 4). Fifth, the inner update *F*_in_ and the recovery update *G* are specified for the subthreshold oscillations and recovery modulation (Step 5).

In the notation below, *v*_*n*_ denotes the discrete membrane-potential variable, *z*_*n*_ the auxiliary recovery variable, and *I* the applied current; (*v*_0_, *n*_0_) denotes the resting equilibrium about which the inner region is centered; *I*_low_ and *I*_high_ denote the lower and upper current levels of the bistable window; *k*_*r*0_ and *k*_*r*1_ mark the transition points used in the outer spiking loop; and ε≪1 is the time-scale-separation parameter that sets the rate of the slow nullcline-guided drift.

**Step 1: Nullcline and slow-branch drift**.

The *v*-nullcline is (Equation 7):


$$\begin{array}{l} f\left(V\right)=\frac{I-g_{l}\left(V-E_{l}\right)-g_{Na}m_{\infty }\left(V\right)\left(V-E_{Na}\right)}{g_{K}\left(V-E_{K}\right)}, \end{array}$$ (7)

with the steady-state activation functions (Equation 8):


$$\begin{array}{l} m_{\infty }\left(V\right)=\frac{1}{1+\exp\left(-\frac{V-v_{hm}}{k_{m}}\right)},\;n_{\infty }\left(V\right)=\frac{1}{1+\exp\left(-\frac{V-v_{hn}}{k_{n}}\right)}. \end{array}$$ (8)

Applying standard nullcline analysis and slow-fast reduction to the continuous branches, the reduced slow motion along the *v*-nullcline is (Fenichel, 1979; Rubin and Terman, 2002):


$$\begin{array}{l} \frac{dV}{dt}\approx \varepsilon \frac{n_{\infty }\left(V\right)-f\left(V\right)}{f^{\prime }\left(V\right)}. \end{array}$$ (9)

On the left wing, where *n*_∞_(*V*) ≈ 0: *dV*/*dt* ≈ −ε*f*(*V*)/*f*′(*V*); on the right wing, where *n*_∞_(*V*) ≈ 1: *dV*/*dt* ≈ ε(1−*f*(*V*))/*f*′(*V*). Thus, the construction uses two standard components of geometric neuronal dynamics: phase-plane nullcline analysis to identify the resting branch, knees, and bistable structure, and slow-fast reduction to approximate motion along the attracting branches of the *v*-nullcline (Fenichel, 1979; Rinzel and Ermentrout, 1989; Rubin and Terman, 2002; Ermentrout and Terman, 2010a).

**Step 2: Outer-loop voltage bounds**.

The outer loop is bounded by *v*_low_ and *v*_high_, estimated from the limiting conductance balances of the continuous model:


$$\begin{array}{l} g_{l}\left(v_{\text{low}}-E_{l}\right)+g_{K}\left(v_{\text{low}}-E_{K}\right)=I, \end{array}$$ (10)

$$\begin{array}{l} g_{l}\left(v_{\text{high}}-E_{l}\right)+g_{Na}m_{\infty }\left(v_{\text{high}}\right)\left(v_{\text{high}}-E_{Na}\right)=I. \end{array}$$ (11)

**Step 3: Current-dependent inner switching region**.

The size of the inner region is controlled by the applied current. Let *I*_low_ and *I*_high_ denote the current levels at which the unstable limit cycle appears and disappears as *I* increases through the bistable window. Define:


$$\begin{array}{l} s\left(I\right)=\max\left(0,\frac{I_{\text{high}}-I}{I_{\text{high}}-I_{\text{low}}}\right),\;\delta_{v}\left(I\right)=8s\left(I\right), \end{array}$$ (12)

so that the voltage width of the inner region shrinks as *I* approaches the Hopf point. The inner switching region is:


$$\begin{array}{l} \Omega_{\text{in}}\left(I\right)=\left\{\left(v,z\right):|v-v_{0}|\leq 3\delta_{v}\left(I\right),n_{0}\leq z\leq 6n_{0}\right\}. \end{array}$$ (13)

**Step 4: Switching structure and outer spiking update**.

The discrete system is (Equation 14):


$$\begin{array}{l} v_{n+1}=F\left(v_{n},z_{n}\right),\;z_{n+1}=G\left(v_{n},z_{n}\right), \end{array}$$ (14)

with the switching rule (Equation 15):


$$F(v,z)=\{\begin{array}{ll} F_{\text{in}}(v,z), & (v,z)\in \Omega_{\text{in}}(I),\\ F_{\text{out}}(v), & (v,z)\notin \Omega_{\text{in}}(I). \end{array}$$ (15)

Outside the inner region, the iterate follows the outer spiking loop:


$$F_{\text{out}}(v)=\{\begin{array}{ll} v-\varepsilon \frac{f(v)}{f^{\prime }(v)}, & v_{\text{low}}\leq v<k_{r0},\\ 0.01v+v_{\text{high}}, & k_{r0}\leq v<k_{r1},\\ v_{\text{low}}+0.001v, & k_{r1}\leq v<k_{r1}+10,\\ v-\varepsilon \frac{1-f(v)}{f^{\prime }(v)}, & k_{r1}+10\leq v\leq v_{\text{high}}. \end{array}$$ (16)

The first and fourth branches represent slow nullcline-guided drift along the left and right wings respectively; the middle two branches produce the fast upstroke and downstroke, as in Zeki and Kapçak (2023).

**Step 5: Inner focus update and recovery variable**.

Inside Ω_in_(*I*), the voltage update is:


$$\begin{array}{l} \;F_{\text{in}}(v,z)\\ =\{\begin{array}{ll} v-\varepsilon \frac{f(v)}{f^{\prime }(v)}, & v_{0}-3\delta_{v}\leq v<v_{0}-0.2\delta_{v},\\ v_{0}+20(z-n_{0})+0.01v, & v_{0}-0.2\delta_{v}\leq v<v_{0},\\ v_{0}-20(z-n_{0})+0.01v, & v_{0}\leq v<v_{0}+0.2\delta_{v},\\ v+\varepsilon \frac{f(v)}{f^{\prime }(v)}, & v_{0}+0.2\delta_{v}\leq v\leq v_{0}+3\delta_{v}. \end{array} \end{array}$$ (17)

This inner map generates the local subthreshold oscillations characteristic of focus dynamics near the Hopf equilibrium.

The auxiliary variable is updated by:


$$G(v,z)=\{\begin{array}{ll} z-\varepsilon 1_{\left\{z>n_{0}\right\}}, & (v,z)\in \Omega_{\text{in}}(I),\\ z-\varepsilon , & (v,z)\notin \Omega_{\text{in}}(I)\text{ and }v<k_{r1},\\ 1, & (v,z)\notin \Omega_{\text{in}}(I)\text{ and }v\geq k_{r1}. \end{array}$$ (18)

Thus *z*_*n*_ decreases gradually in the inner region and during the left part of the outer loop, and is reset after the upstroke, simulating the slow activation and decay of recovery currents.

Table 2 summarizes which components of the construction are guided by the nullcline geometry of the continuous model and which are phenomenological reduced-model choices.

**Table 2:** Classification of the discrete-map construction components as nullcline-guided or phenomenological.

Component/constant	Equations	Type
Slow-drift branches	Equation 9	Nullcline-guided
Outer bounds *v*_low_, *v*_high_	Equations 10, 11	Nullcline-guided
Resting equilibrium (*v*_0_, *n*_0_)	—	Nullcline-guided
Inner-region width δ_*v*_ = 8*s*(*I*)	Equation 12	Phenomenological
Region multiples 3δ_*v*_, 6*n*_0_	Equation 13	Phenomenological
Switching half-width 0.2δ_*v*_	Equation 17	Phenomenological
Focus switching coefficient 20	Equation 17	Phenomenological
Shaping slopes 0.01, 0.001	Equations 16, 17	Phenomenological

Nullcline-guided components inherit their parameter dependence from the continuous *I*_*Na, p*_+*I*_*K*_ model; phenomenological components are reduced-model choices fixed so that the inner region reproduces a damped focus-like return and shrinks as *I* approaches the Hopf threshold.

The damped subthreshold return in the discrete map is produced by the piecewise switching structure inside Ω_in_(*I*) rather than by a single smooth linearisation at the resting point. Away from the switching boundaries, the two central branches of *F*_in_ in Equation 17 have the branch-wise linear parts


$$D_{-}=\left(\begin{array}{cc} 0.01 & \kappa \\ 0 & 1 \end{array}\right),\;D_{+}=\left(\begin{array}{cc} 0.01 & -\kappa \\ 0 & 1 \end{array}\right),\;\kappa \approx 58,$$

with κ set by the inner-region geometry. Each branch is upper triangular with real multipliers {0.01, 1}, so no individual branch is a focus. The oscillation arises from the alternation of *D*_−_ and *D*_+_: the coupling to the recovery variable has opposite sign on the two sides of the resting point, so successive iterates are thrown to alternating sides of *v*_0_, while the recovery update *G* in Equation 18 contracts the excursion amplitude through a slow drift in *z*_*n*_. The result is a damped sequence of alternating subthreshold excursions that reproduces the damped subthreshold return of the continuous focus, without constituting a smooth-map bifurcation in the formal sense.

Because the map is piecewise-defined, this local-return mechanism should not be interpreted as a formal Neimark–Sacker bifurcation of a smooth discrete map. Instead, the construction encodes the same stability geometry identified in the continuous system: return to rest through damped subthreshold oscillations inside Ω_in_(*I*), capture by the outer spiking loop *F*_out_ outside this region, and contraction of the inner region as δ_*v*_(*I*) decreases near the Hopf threshold.

#### 2.4.1 Validity of the slow-manifold approximation and fold points

The slow-branch drift increments are motivated by the standard slow–fast reduction of the continuous model near the attracting branches of the *v*-nullcline, with ε setting the rate of the nullcline-guided drift. In all simulations reported here we use ε = 0.5.

The reduction in Equation 9 is valid only on regular portions of the slow branches. On compact subintervals of an attracting branch where the critical manifold is normally hyperbolic and |*f*′(*V*)|≥*m*>0, Fenichel theory guarantees that the perturbed slow manifold lies within *O*(ε) of the critical manifold, with constants depending on the lower bound *m*. The slow-drift expression should therefore be read as a regular-branch approximation rather than a formula valid uniformly over the whole nullcline. As the fold points (knees) of the *v*-nullcline are approached, *f*′(*V*) → 0, normal hyperbolicity is lost, and the increment ε*f*(*V*)/*f*′(*V*) ceases to be a meaningful slow-manifold approximation.

The map is constructed so that it is never iterated through a fold using this rule. The slow-drift branches of *F*_out_ in Equation 16 are defined only up to the switching thresholds *k*_*r*0_ and *k*_*r*1_, which are placed at the knees of the *v*-nullcline. When a trajectory drifting along a slow branch reaches the corresponding threshold, the map leaves the nullcline-guided rule and switches to the phenomenological fast upstroke or downstroke branch; it does not continue to evaluate the slow-drift increment in the singular fold neighborhood beyond the knee. The fold points are thus handled by the piecewise switching structure of the map rather than by a uniformly valid slow-manifold approximation, and the passage through each fold is represented by the fast branch rather than resolved in detail.

This is an intentional reduction and a stated limitation. The map is designed to preserve the large-scale dynamical organization of the subcritical-Hopf regime—slow return, bistability, hysteresis, and escape to the outer spiking loop—but not to reproduce the detailed continuous-time passage through the fold neighborhoods.

#### 2.4.2 Numerical implementation and reproducibility

All simulations were implemented in MATLAB. The continuous *I*_*Na, p*_+*I*_*K*_ model was simulated by direct numerical integration using MATLAB's ode45 solver, an adaptive explicit Runge–Kutta method based on the Dormand–Prince pair. Unless otherwise stated, the default relative and absolute tolerances, 10^−3^ and 10^−6^, were used. The continuous phase-plane figures were generated from representative initial conditions inside and outside the unstable limit cycle, and the nullclines were plotted directly from their defining equations.

The discrete map itself does not require an ODE solver. It is evaluated by explicit algebraic iteration of the piecewise difference equations. At each step, the state (*v*_*n*_, *z*_*n*_) is tested for membership in Ω_in_(*I*). If the state lies inside this region, the update *F*_in_ is applied; otherwise, the update *F*_out_ is applied. The recovery variable is then updated using *G*. Cobweb diagrams were generated from the pairs (*v*_*n*_, *v*_*n*+1_), while time-series panels were generated by plotting *v*_*n*_ against the iteration index *n*.

The conceptual prototype was simulated by direct iteration of Equation 6. Period-2 points were identified from intersections of *f*(*x*) with the line *y* = −*x*, and their stability was classified using the multiplier of the second iterate. For the hysteresis and resonance simulations, the applied current was prescribed externally as described in the corresponding figure captions. Unless otherwise stated, all other model parameters were kept fixed at the values given in the model definition and figure captions.

### 2.5 Computational benchmarking protocol

To substantiate the computational motivation for the proposed discrete map, we benchmarked trajectory generation against explicit Euler and fourth-order Runge–Kutta (RK4) integration of the corresponding continuous *I*_Na, p_+*I*_*K*_ system. The comparison was designed as an implementation-level timing test rather than a pointwise dynamical equivalence test. In particular, one iteration of the proposed map is not assumed to correspond to one numerical integration step of the continuous ODE. Instead, all methods were required to produce the same number of saved trajectory states.

For each method, two initial conditions were simulated for 8,000 saved updates per initial condition, giving 16,000 saved state updates in total. Euler and RK4 were run with Δ*t* = 0.05 for the timing comparison. RK4 therefore used four right-hand-side evaluations per saved state, whereas Euler and the discrete map used one update per saved state. Timing was repeated 20 times, and the median runtime was reported. Plotting, figure export, and file-writing operations were excluded from all timing measurements.

Because the present manuscript develops a single-cell map and does not yet specify a synaptically coupled network architecture, we report normalized cost per saved state update rather than claiming a network-level speedup. For uncoupled cells, the total cost scales linearly with the number of cells and the number of saved updates; for coupled networks, additional synaptic-update costs would have to be benchmarked separately.

We also distinguished between the original scalar implementation of the map and an algebraically equivalent optimized scalar implementation. The optimized version removes repeated interval classification and unused computations while preserving the map output exactly. Agreement between the original and optimized implementations was verified by comparing voltage trajectories, recovery-variable trajectories, and map-curve values; the maximum absolute difference was zero in all checks.

## 3 Results

The discrete-time dynamical system derived in Section 2.4 successfully reproduces the three hallmark behaviors of a neuron governed by a subcritical Hopf bifurcation: bistable resting and spiking dynamics, hysteresis with respect to the injected current, and frequency-selective resonance. Each property is examined in turn below.

### 3.1 Bistability and map dynamics

**Figure 7:**
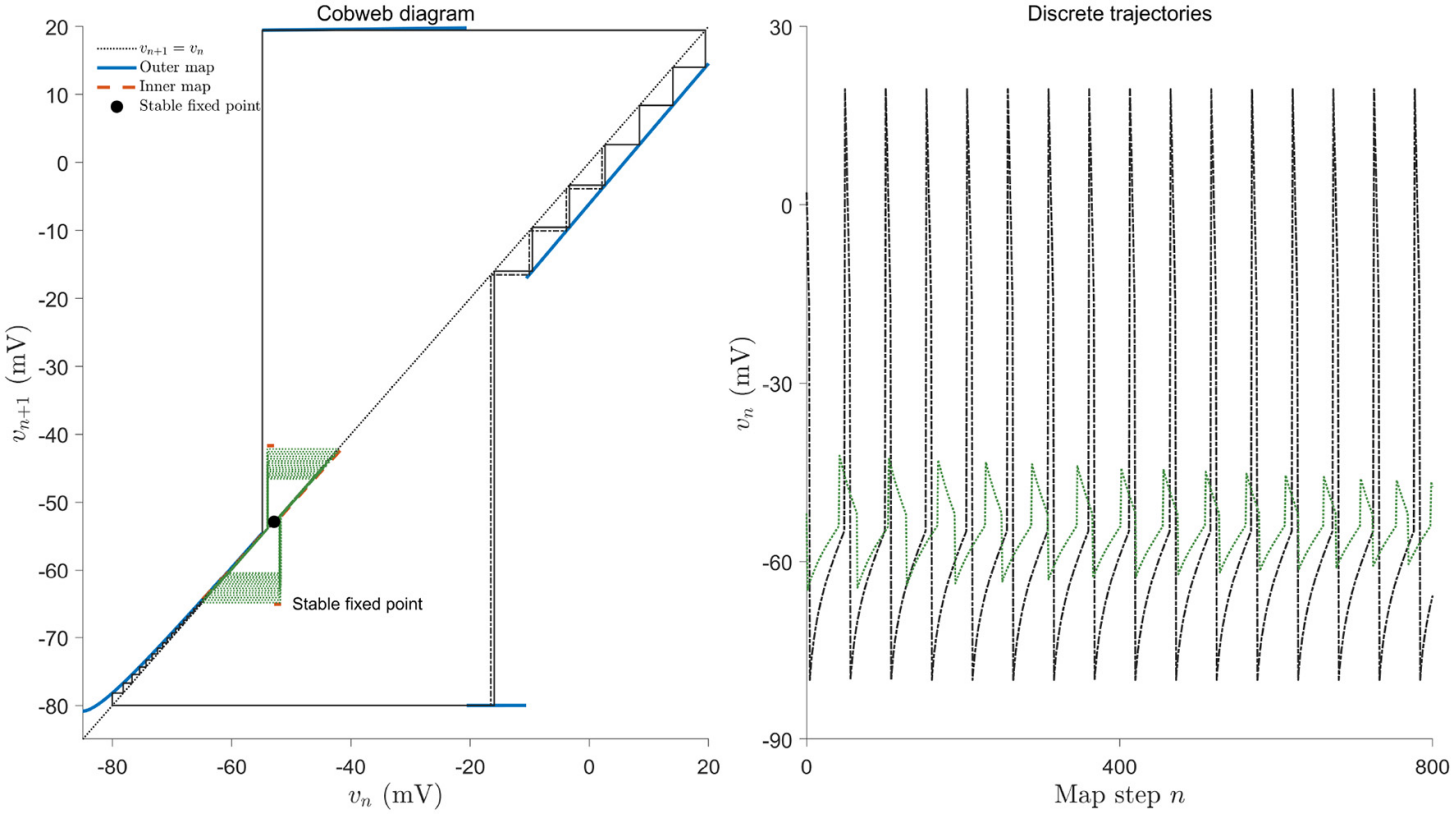
Bistable dynamics of the full discrete map at *I* = 34μA. The map uses the same conductance and activation parameters as the continuous model, with the current-dependent inner region Ω_in_(*I*) defined by δ_*v*_(*I*). **(Left)**, cobweb diagram showing coexistence of a stable fixed point (black dot) and a large-amplitude stable periodic orbit. Green trajectories are initiated inside Ω_in_(*I*) and return to rest through damped subthreshold oscillations governed by *F*_in_. Black trajectories are initiated outside Ω_in_(*I*) and are captured by the outer spiking loop *F*_out_. **(Right)**, time series over 800 map steps showing subthreshold return and sustained spiking for the two initial conditions.

Figure 7 illustrates the core dynamical structure of the discrete map at *I* = 34μA (Ω_in_≠∅). The cobweb diagram on the left reveals two coexisting attractors: a stable fixed point near *v*_*n*_ ≈ −53mV (black dot), corresponding to the resting state, and a large-amplitude stable periodic orbit (“Stable periodic solution”), corresponding to repetitive spiking. Separating them is the inner region Ω_in_, analogous to the ULC of the continuous model (cf. Figure 1). Trajectories initiated inside Ω_in_ (green) are governed by *F*_in_ (Equation 17) and spiral inward toward the fixed point, producing damped subthreshold oscillations that replicate the focus-like behavior of the continuous *I*_*Na, p*_+*I*_*K*_ model. Trajectories outside (black) are governed by *F*_out_ (Equation 16) and produce relaxation-style action potentials with a sharp upstroke near *v*_*n*_ ≈ 20mV and a hyperpolarisation trough near *v*_*n*_ ≈ −80mV.

The recovery variable *z*_*n*_ (Equation 18) modulates the width of Ω_in_ across cycles, so the damped subthreshold oscillations (green, right panel) exhibit a gradually decaying amplitude envelope before settling to the fixed point. The cobweb structure confirms that the discrete map captures the bistable phenomenology of the subcritical Hopf bifurcation within a computationally tractable framework.

### 3.2 Hysteresis

**Figure 8:**
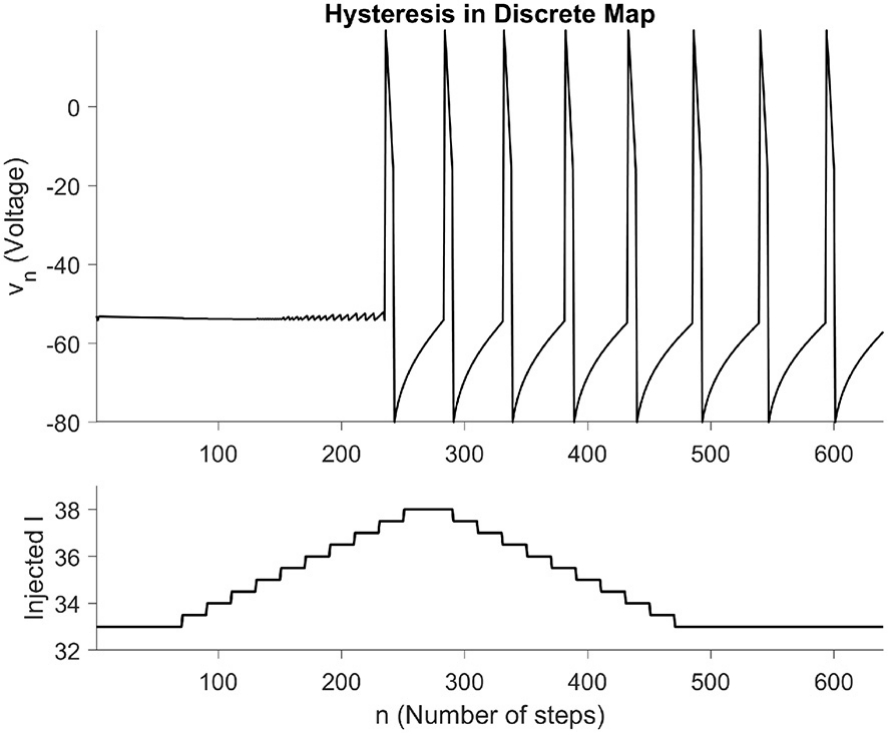
Hysteresis in the full discrete map. Upper panel: membrane potential *v*_*n*_ as a function of map step *n*. Lower panel: applied-current profile. The current is ramped from approximately 33μA to 38μA and then decreased back toward baseline. As *I* crosses the bifurcation threshold, δ_*v*_(*I*) decreases and the inner region Ω_in_(*I*) collapses, causing the trajectory to escape to the outer spiking loop. Spiking persists during the descending current ramp, demonstrating hysteresis in the discrete model.

A defining consequence of bistability in the subcritical Hopf regime is hysteresis: the state of the neuron depends not only on the current value of *I* but also on its history. Figure 8 demonstrates this in the discrete map, reproducing the behavior of the continuous model (cf. Figure 2). The injected current is ramped upward from *I* ≈ 33μA in discrete steps to a peak of *I* ≈ 38μA and then decreased symmetrically back to baseline.

During the ascending phase (*n* < 200), the neuron remains in the stable resting state, exhibiting small-amplitude subthreshold fluctuations governed by *F*_in_ within a wide Ω_in_. As *I* crosses the bifurcation threshold — where δ_*v*_(*I*) → 0 and Ω_in_ collapses — the resting state loses stability and the trajectory is abruptly captured by the outer spiking loop, producing a sudden onset of large-amplitude action potentials near *n* ≈ 230. When the current is subsequently reduced, spiking persists well below the level at which it was initiated. The neuron remains locked to the spiking orbit for the remainder of the simulation, demonstrating the path-dependent behavior that is the hallmark of hysteresis (Izhikevich, 2007).

### 3.3 Resonance

Because trajectories inside Ω_in_ return to rest through damped, amplitude-dependent subthreshold oscillations over a characteristic recovery timescale, the discrete map exhibits resonance, in agreement with the continuous model (cf. Figure 3) and with the theoretical predictions for subcritical Hopf neurons (Hutcheon and Yarom, 2000; Izhikevich, 2001).

#### 3.3.1 Intrinsic period diagnostic

**Figure 9:**
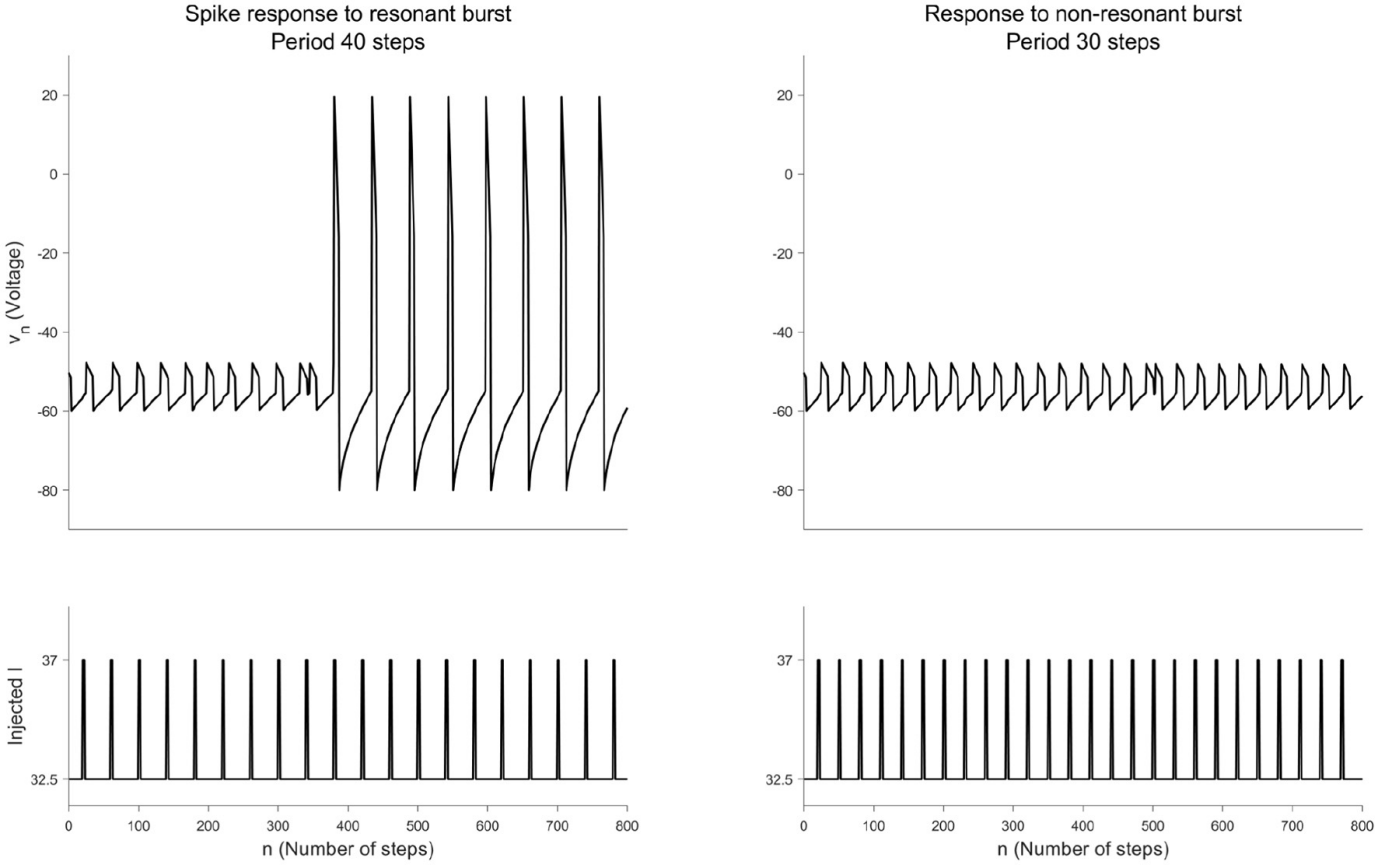
Frequency-selective resonance in the full discrete map. The applied current alternates between *I* = 32.5μA and *I* = 37μA in both cases; only the burst period is changed. **(Left)**, period-40 burst stimulation arrives near the constructive phase of the subthreshold return and leads to progressive amplification, escape from Ω_in_(*I*) near *n* ≈ 380, and sustained spiking. **(Right)** period-30 burst stimulation arrives off phase and fails to produce constructive amplification, so the trajectory remains subthreshold. Upper panels show membrane potential *v*_*n*_; lower panels show the injected-current protocol.

A perturbation of the resting state elicits a damped subthreshold oscillation whose amplitude decays toward *v*_0_ over the recovery timescale set by *G*. Its period is not constant: the large-amplitude cycles are slower and the oscillation accelerates as it decays toward rest, so the response is characterized by a dominant large-amplitude timescale rather than a single fixed frequency. The resonant and non-resonant burst periods in Figure 9 were chosen relative to this dominant subthreshold timescale.

Figure 9 compares the response to two periodic burst inputs alternating between *I* = 32.5μA and *I* = 37μA. Under the resonant stimulus (period 40 steps, left column), each pulse arrives as the membrane potential is on an upward phase of its damped oscillation, so successive inputs constructively amplify the excursion. Starting from subthreshold oscillations near −60mV, the membrane potential builds across cycles until it breaches Ω_in_ near *n* ≈ 380 and is captured by *F*_out_, triggering a train of full-amplitude action potentials.

Under the non-resonant stimulus (period 30 steps, right column), successive pulses arrive out of phase with the intrinsic oscillation and do not constructively accumulate. The membrane potential remains confined to small-amplitude oscillations near −60mV throughout the entire 800-step simulation.

This frequency selectivity arises from the switching mechanism embedded in *F*_in_ (Equation 17): the sign reversal of the recovery coupling across *v*_0_ produces alternating subthreshold excursions whose dominant timescale is set by the recovery drift in *G*, not by a fixed eigenfrequency. Because this timescale is inherited from the same inner-region geometry that the nullcline-guided bounds constrain, the discrete map preserves the resonator property that fundamentally distinguishes subcritical Hopf neurons from integrator-type cells (Izhikevich, 2000; Hutcheon and Yarom, 2000).

### 3.4 Feature-level validation against the continuous model

The proposed map is a reduced, piecewise discrete representation of the continuous *I*_*Na, p*_+*I*_*K*_ model, not a numerical time-discretisation of the ordinary differential equations. Therefore, pointwise voltage-trace errors such as RMSE or MAE are not the primary validation criterion. Such metrics would implicitly assume a one-to-one correspondence between continuous time and map iteration number and would penalize the intended waveform simplifications of the reduced map.

Instead, validation is performed at the level of the dynamical features that define subcritical Hopf excitability. The target features are: (i) coexistence of a stable resting state and a stable spiking orbit, separated by an unstable boundary; (ii) loss of stability of the resting equilibrium as the applied current is increased; (iii) hysteresis under a slowly varying current; and (iv) frequency-selective amplification of inputs delivered at a constructive phase of the subthreshold return.

For the continuous reference model, the change of stability is verified by the Jacobian eigenvalue calculation of Equation 4: the resting equilibrium is a stable focus at *I* = 34 and 35μA and loses stability through the subcritical Hopf bifurcation at *I*_Hopf_ ≈ 35.4μA. In the discrete map the corresponding structure is represented by the switching geometry: trajectories inside Ω_in_(*I*) return to rest through damped focus-like oscillations governed by *F*_in_, whereas trajectories outside Ω_in_(*I*) are captured by the outer spiking loop *F*_out_. The current dependence enters through δ_*v*_(*I*) (Equation 12), which contracts the inner region as the current increases; Ω_in_(*I*) collapses and the resting branch disappears at *I* ≈ 36μA, within about 1.7% of the continuous Hopf current. This close agreement reflects in part that the inner-region current window was chosen consistently with the continuous bifurcation, rather than emerging from the map independently.

For the resonance comparison, the relevant discrete quantity is not a single linear eigenfrequency at the resting point. The inner map is piecewise-defined, and trajectories inside Ω_in_(*I*) pass through different branches of *F*_in_ while the recovery variable *z*_*n*_ evolves under *G*. Consequently, the subthreshold return is a damped, amplitude- and phase-dependent trajectory rather than a sinusoidal oscillation with one constant period: its small-amplitude limit is short (on the order of five steps) and lengthens at larger amplitude. Under the identification of one map step with approximately one millisecond, this small-amplitude limit is close to the continuous focus eigenperiod (≈ 4.6ms, from Imλ ≈ 1.37ms^−1^). In Figure 9, the period-40 burst is a constructive stimulation protocol for this damped return, whereas the period-30 burst is an off-phase protocol that fails to accumulate sufficiently to trigger escape to the outer spiking loop.

A direct visual comparison of the two systems is provided by the continuous-model phase portraits and trajectories (Figures 1–3) and the corresponding discrete-map trajectories (Figures 7–9), which display the same bistable, hysteretic, and resonant behavior. Table 3 summarizes the comparison.

**Table 3:** Feature-level validation of the proposed discrete map against the continuous *I*_*Na, p*_+*I*_*K*_ reference model.

Target feature	Continuous *I*_*Na, p*_+*I*_*K*_ model	Discrete map
Resting-state stability	Stable focus at *I* = 34, 35μA; loss of stability from the Jacobian eigenvalues	Damped subthreshold return inside Ω_in_(*I*), generated by *F*_in_ and the recovery update *G*
Bifurcation threshold	*I*_Hopf_ ≈ 35.4μA	Ω_in_ collapses / resting branch lost at *I* ≈ 36μA (≈ 1.7% difference)
Bistability	Stable resting focus coexists with a stable spiking limit cycle, separated by an unstable limit cycle	Trajectories inside Ω_in_(*I*) return to rest; those outside are captured by the outer spiking loop *F*_out_
Hysteresis	Remains in the oscillatory state after the current is reduced, due to coexisting attractors	Trajectory remains on the outer spiking loop during the descending ramp after escape
Subthreshold oscillation period	≈ 4.6ms (Imλ ≈ 1.37/ms)	≈ 5 steps at small amplitude (≈ 5ms at 1 step ≈ 1ms); amplitude-dependent
Frequency selectivity	A 7ms stimulus period elicits a spike; an off-resonant period does not	A period-40 burst is constructive and triggers spiking; a period-30 burst remains subthreshold

The comparison is made at the level of dynamical structure rather than pointwise voltage-trace error, because the map is a reduced piecewise representation rather than an ODE time-stepper. One discrete step is used as a nominal protocol-level unit of approximately one millisecond for stimulation-period comparisons; it is not an ODE time-discretisation.

This feature-level comparison supports the intended claim of the model: the discrete map preserves the qualitative bifurcation structure and dynamical signatures of the continuous subcritical-Hopf system. It does not imply that the map reproduces the continuous voltage trace point by point.

### 3.5 Scope of parameter variation

The principal parameter varied in this study is the applied current *I*, which is the bifurcation parameter of the continuous *I*_*Na, p*_+*I*_*K*_ model and also the control parameter of the discrete map. The current is varied across the bistable window *I*∈[32, 36]μA, within which the continuous model passes through the subcritical Hopf bifurcation at *I*_Hopf_ ≈ 35.4μA, and the discrete map is driven over the same range. Varying *I* is therefore the dynamically meaningful way to test whether the construction reproduces the sequence of regimes associated with subcritical Hopf excitability: coexistence of resting and spiking states, loss of resting-state stability, hysteresis, and frequency-selective response. Within this window the individual regimes are illustrated at representative currents, while the hysteresis result sweeps *I* continuously across the window. The reported behaviors are thus obtained across the relevant range of the biologically central bifurcation parameter, not at a single fixed value.

This bifurcation-parameter variation should be distinguished from an unrestricted sensitivity analysis over all model parameters. The conductances, reversal potentials, nullcline geometry, fast-branch slopes, and the numerical factors defining Ω_in_(*I*) are calibration parameters that fix the model to the subcritical-Hopf regime rather than free parameters to be swept independently: together they determine whether the continuous system lies in a subcritical-Hopf regime and whether the discrete map is matched to that phase-plane geometry. We verified that the reported behaviors persist under modest variation of the principal parameters within the Hopf window. A systematic multidimensional study, however, would require re-identifying the Hopf window and recalibrating the map for each parameter combination, and is left as future work.

In the continuous model, increasing *I* changes the stability of the resting equilibrium and the relative position of the unstable and stable limit cycles. In the discrete map, the same parameter sets the width of the inner region Ω_in_(*I*) through δ_*v*_(*I*) in Equation 12, regulating whether trajectories remain in the subthreshold focus-like region or escape to the outer spiking loop.

### 3.6 Computational benchmark against Euler and RK4

The trajectory-generation benchmark is summarized in Table 4. The original scalar MATLAB implementation of the discrete map required 4.34μs per saved state update. After algebraically equivalent optimization, the same map required 0.62μs per saved state update. Thus, the optimized implementation was approximately 7.0-fold faster than the original scalar implementation.

**Table 4:** Trajectory-generation runtime benchmark for the discrete map and continuous ODE solvers.

Method	Time (s)	Updates	Calls	μs/update	Rel.
Original map	0.06937	16,000	16,000	4.34	7.04
Optimized map	0.00985	16,000	16,000	0.62	1.00
Euler ODE	0.00757	16,000	16,000	0.47	0.77
RK4 ODE	0.02905	16,000	64,000	1.82	2.95

Rel. denotes runtime relative to the optimized discrete-map implementation.

All methods produced 16,000 saved state updates, corresponding to two initial conditions and 8,000 saved updates per initial condition. Euler and RK4 used Δ*t* = 0.05. Timings are medians over 20 repetitions and exclude plotting, export, and file-writing operations.

The optimized map was close in cost to explicit Euler integration and substantially faster than RK4 under the same saved-update benchmark. Euler required 0.47μs per saved state update, whereas RK4 required 1.82μs per saved state update. Therefore, the optimized map was approximately 1.30-fold slower than Euler but approximately 2.95-fold faster than RK4. This result supports the computational lightness of the proposed map relative to higher-order continuous-time integration, while also showing that the map is not simply a faster replacement for every ODE solver. Rather, it is a reduced phase-plane-guided discrete system whose computational cost is comparable to a simple explicit method and lower than RK4 in the present implementation.

As a separate implementation check, the map-curve calculation used for plotting was also vectorized. This reduced the time for generating the inner and outer map curves from 5.38 × 10^−2^ s to 1.54 × 10^−3^ s, corresponding to an approximately 35-fold improvement. This plotting-related benchmark was not included in the trajectory-generation timings in Table 4; it is reported only to document that the discrete map can be evaluated efficiently over independent voltage grids.

## 4 Discussion

The central contribution of this work is a discrete-time map that captures bistability, hysteresis, and frequency-selective resonance in a subcritical Hopf neuron, within a piecewise, parameter-preserving framework. We discuss the approach relative to existing discrete and reduced models, assess its limitations, and outline directions for further development.

### 4.1 Relation to existing discrete-time approaches

Discrete-time representations of neural dynamics fall broadly into two families. The first consists of Poincaré-section maps, in which a continuous periodic orbit is sampled at a fixed phase and the resulting return map is used to study synchronization (Channell et al., 2007; Terman et al., 2008). These maps are exact in the periodic regime but are undefined for transient or subthreshold trajectories and carry no information about waveform shape between samples. The present model does not rely on periodicity: the map is defined over the entire voltage range, represents the full cycle from hyperpolarisation through spike to reset, and produces damped oscillatory transients in the resting basin. This is the same conceptual advantage that distinguished our earlier SNIC map (Zeki and Kapçak, 2023) from Poincaré-based approaches, and it extends directly to the subcritical Hopf setting.

#### 4.1.1 Advantages and scope of the nullcline-guided construction

The nullcline-guided interpretation occupies an intermediate level of description between direct numerical integration of the continuous conductance-based model and a fully phenomenological discrete map, and this intermediate position is its main advantage. Because the slow-branch increments follow the reduced drift along the wings of the *v*-nullcline (Equation 9) and the outer-loop bounds *v*_low_ and *v*_high_ are defined by the same limiting conductance balances as the continuous model (Equations 10–11), the discrete dynamics inherit the parameter dependence of the original system rather than approximating it. For example, increasing the potassium conductance *g*_*K*_ reshapes the nullcline function *f*(*V*) and shifts *v*_low_ and *v*_high_ through the very balances that define them, so the qualitative effect of a biophysical change can be anticipated without re-fitting the map. Phenomenological maps such as those of Chialvo (1995) and Rulkov (2002) cannot offer this, since their coefficients bear no fixed relation to conductances or reversal potentials. Moreover, this link to the underlying ion-channel dynamics is retained without integrating a differential equation at any step, keeping the construction computationally light.

The scope of this guidance should nonetheless be stated plainly, as noted in the Introduction: it applies most directly to the slow branches and the outer-loop bounds, whereas the fast-jump branches, the inner switching region, and selected waveform-shaping coefficients remain phenomenological. The map is therefore best understood as a nullcline-guided reduced representation rather than an exact or fully parameter-free discretisation of the continuous system.

#### 4.1.2 Broader dynamical-systems context

While the present work focuses on deriving a discrete map for neural excitability, the broader analysis of Hopf bifurcations and multistability in nonlinear dynamical systems provides useful context for the construction. Recent studies have examined multistability and a multi-spiral chaotic sea in a three-dimensional system with a line of equilibria (Zaamoune et al., 2025b), a hidden bifurcation and Hopf bifurcation generated through saturated function series (Zaamoune and Tidjani, 2024), and time-delayed feedback control of Hopf bifurcations in multi-scroll chaotic attractors (Zaamoune et al., 2025a). These are not conductance-based neuron reductions, but they share with the present construction a central concern: how a local bifurcation organizes the coexistence of attractors separated by an unstable structure—here, the coexistence of rest and spiking separated by the unstable limit cycle in the subcritical-Hopf regime. The present study addresses this theme in a biophysically motivated setting through a nullcline-guided discrete-time map for the *I*_Na, p_+*I*_*K*_ model.

### 4.2 Relation to the previous nullcline-guided map

The present work extends the nullcline-guided framework of Zeki and Kapçak (2023) from type-I (SNIC) to type-II (subcritical Hopf) excitability. The two maps share the same outer spiking loop, whose slow branches are governed by the same nullcline-drift approximation on the left and right wings of the cubic *v*-nullcline (Fenichel, 1979; Rubin and Terman, 2002). The essential new element is the inner switching region Ω_in_, which replaces the simple saddle-node threshold of the SNIC map with a current-dependent focus structure. Within Ω_in_, the map generates damped oscillations rather than monotone convergence to rest, and the width of this region shrinks as *I* increases, encoding the contraction of the ULC that precedes the subcritical Hopf bifurcation. The recovery variable *z*_*n*_ provides the slow modulation needed to reproduce the gradual amplitude decay of the subthreshold focus.

### 4.3 Bistability, hysteresis, and computational consequences

The coexistence of a stable resting fixed point and a stable spiking periodic orbit is reproduced through the switching structure: for the same *I*, trajectories inside Ω_in_ converge to the fixed point while those outside follow the outer loop. This bistable organization makes hysteresis possible, and Figure 8 confirms that the map preserves this feature. From a computational standpoint, bistability endows the neuron with memory of its recent input history, a property linked to working memory maintenance and persistent activity in cortical circuits (Neymotin et al., 2016; Zeki and Moustafa, 2017).

### 4.4 Resonance and frequency-coded processing

The resonance result (Figure 9) illustrates a more subtle preservation: not merely the asymptotic attractor structure, but the transient return dynamics within the resting basin. Because *F*_in_ produces damped, amplitude-dependent subthreshold oscillations over a characteristic recovery timescale, inputs timed to reinforce those oscillations accumulate across cycles, whereas mistimed inputs interfere destructively. This behavior is the defining property of resonator neurons, observed experimentally in entorhinal cortex, thalamus, inferior olive, and auditory brainstem (Hutcheon and Yarom, 2000), and has been proposed as a mechanism for frequency-selective information transfer in neural circuits (Izhikevich, 2001). The discrete map reproduces this without any additional tuning.

### 4.5 Limitations and future directions

Several aspects of the model warrant further development. First, the present construction covers only the bistable regime; extension to the purely oscillatory regime beyond the Hopf point (where Ω_in_ collapses) is straightforward in principle but has not been validated quantitatively here. Second, the map approximates the slow nullcline drift; no formal convergence theorem relating the discrete iterates to the continuous flow is available, and a rigorous error analysis in the spirit of singular perturbation theory (Fenichel, 1979; Rubin and Terman, 2002) remains to be carried out. Third, the slopes used in the fast-jump branches of *F*_out_ are fixed numerical constants rather than nullcline-derived quantities; replacing these would further tighten the correspondence with the original system.

The computational benchmark clarifies the role of the proposed map. The optimized scalar implementation is not faster than a single explicit Euler step in the present MATLAB implementation, but its per-update cost is close to Euler and substantially lower than RK4. Thus, the computational claim should not be interpreted as a universal speedup over all continuous-time solvers. Instead, the advantage of the map is that it provides a compact, event-free, phase-plane-guided discrete update that reproduces the qualitative structure of subcritical Hopf excitability—bistability, hysteresis, and resonance-like subthreshold dynamics—without solving the continuous ODE at each step. The benchmark therefore supports a more precise conclusion: the map is computationally lightweight and faster than RK4 per saved state update in the tested implementation, while its main contribution remains the construction of an interpretable discrete dynamical system rather than a pointwise numerical integrator.

#### 4.5.1 Practical limitations for network use

The present study establishes the single-cell map and does not yet validate it inside a synaptically coupled network. This distinction is important because, in a heterogeneous network, different neurons may receive different effective synaptic drives and may therefore operate in different dynamical regimes. In the current construction, the applied current *I* controls the size of the inner region through the scale factor *s*(*I*) and the width δ_*v*_(*I*). As *I* approaches *I*_high_, *s*(*I*) → 0 and Ω_in_ collapses; for effective inputs beyond this range, the map is governed by the outer loop *F*_out_, corresponding to the post-Hopf spiking regime. This abrupt switching at Ω_in_ is intended behavior for a single cell traversing its own cycle, consistent with the fold-handling described in Section 3.4; its consequences in a network, where many cells may cross the same boundary together, are a separate question that has not yet been tested under rapidly fluctuating synaptic input.

Conductance-based synaptic input could be incorporated by converting synaptic currents into a state-dependent effective input current. Because the discrete voltage variable *v*_*n*_ retains its physiological scale in millivolts, a synaptic conductance can be included at each map step. We adopt the convention that synaptic currents enter as source terms added to the applied current, in the same way that *I* enters the voltage (Equation 1); with this convention an excitatory conductance (*E*_*E*_>*v*_*n*_) increases the effective drive. With excitatory and inhibitory synaptic conductances *g*_*E*_(*n*) and *g*_*I*_(*n*),


$$I_{\text{eff},n}=I_{\text{app}}+g_{E}\left(n\right)(E_{E}-v_{n})+g_{I}\left(n\right)(E_{I}-v_{n}).$$

The nullcline function, resting equilibrium, knee locations, and inner-region width would then be recomputed quasi-statically from *I*_eff, *n*_, treating it as a slowly varying bifurcation parameter. This quasi-static route is natural when the synaptic variables evolve slowly relative to the recovery timescale; rapidly fluctuating synaptic input would violate the quasi-static assumption and require the inner-region geometry to be re-derived rather than tracked, which introduces new validation requirements because *I*_eff, *n*_ is then voltage- and time-dependent rather than a fixed bifurcation parameter.

Finally, the piecewise nature of the map may introduce network-level artifacts if used without additional smoothing or validation. Abrupt switching at Ω_in_, at the knee thresholds *k*_*r*0_ and *k*_*r*1_, or between the fast and slow branches could produce artificial phase locking, chattering near switching surfaces, or enhanced synchronization in populations of nearly identical neurons: if many cells cross the same switching boundary simultaneously, the piecewise rule can suppress the smooth dispersion of phases and spike times that a continuous stochastic network would produce. Practical network implementations may therefore require smoothed switching functions, hysteretic boundary rules, noise terms, or refractory constraints. A systematic comparison between coupled networks of the discrete map and coupled networks of the continuous *I*_Na, p_+*I*_*K*_ model is an important direction for future work.

#### 4.5.2 Biological interpretation and validation scope

The proposed map should be interpreted as a theoretical and computational reduced model rather than as a directly experimentally validated biological neuron model. Its biological grounding comes from the continuous *I*_*Na, p*_+*I*_*K*_ conductance-based reference system, whose variables and parameters carry standard electrophysiological meanings, and from the well-established association between subcritical Hopf (Class 2) excitability, bistability, hysteresis, and subthreshold resonance (Izhikevich, 2007; Hutcheon and Yarom, 2000). The agreement of the discrete map with these subcritical-Hopf dynamics of the continuous reference model is established in the Results (Figures 7–9).

What the present study does not claim is that the map has been directly fitted to, or validated against, experimental *in vitro* or *in vivo* neural recordings from a specific cell type. Its biological realism is therefore indirect: the map retains selected conductance-based parameters and phase-plane structures of the continuous model, while the piecewise switching rules, inner-region geometry, and waveform-shaping coefficients remain reduced-model components. Experimental calibration and validation against recorded resonator neurons—such as cells in the thalamus, entorhinal cortex, inferior olive, or auditory brainstem (Hutcheon and Yarom, 2000)—remain important directions for future work.

## Data Availability

The original contributions presented in the study are included in the article/supplementary material, further inquiries can be directed to the corresponding author.
